# Does AMPK bind glycogen in skeletal muscle or is the relationship correlative?

**DOI:** 10.1042/EBC20240006

**Published:** 2024-11-18

**Authors:** Barnaby P. Frankish, Robyn M. Murphy

**Affiliations:** 1 Sport, Exercise and Nutrition Sciences, School of Allied Health, Human Services and Sport, La Trobe University, Melbourne, VIC 3086, Australia; 2 Department of Biochemistry and Chemistry, La Trobe Institute for Molecular Science, School of Agriculture, Biomedicine and Environment, La Trobe University, Melbourne, VIC 3086, Australia Insert Affiliation Text Here.

**Keywords:** AMPK, glycogen, skeletal muscle

## Abstract

Since its discovery over five decades ago, an emphasis on better understanding the structure and functional role of AMPK has been prevalent. In that time, the role of AMPK as a heterotrimeric enzyme that senses the energy state of various cell types has been established. Skeletal muscle is a dynamic, plastic tissue that adapts to both functional and metabolic demands of the human body, such as muscle contraction or exercise. With a deliberate focus on AMPK in skeletal muscle, this review places a physiological lens to the association of AMPK and glycogen that has been established biochemically. It discusses that, to date, no *in vivo* association of AMPK with glycogen has been shown and this is not altered with interventions, either by physiological or biochemical utilisation of glycogen in skeletal muscle. The reason for this is likely due to the persistent phosphorylation of Thr148 in the β-subunit of AMPK which prevents AMPK from binding to carbohydrate domains. This review presents the correlative data that suggests AMPK senses glycogen utilisation through a direct interaction with glycogen, the biochemical data showing that AMPK can bind carbohydrate *in vitro*, and highlights that in a physiological setting of rodent skeletal muscle, AMPK does not directly bind to glycogen.

## AMP-activated protein kinase (AMPK)

The 5′-AMP-activated protein kinase (AMPK) is a crucial enzyme that functions as an intracellular fuel sensor and hence plays a significant role in cellular energy homeostasis and physiological adaptations. It is most highly expressed in energy consuming and producing tissues, such as liver, adipose tissue, pancreatic β cells, brain, heart and skeletal muscle, but it is also present in other tissues [1,2].

In skeletal muscle, AMPK is activated in response to exercise and energy utilisation and its activity is typically highest during contraction when glycogen is being utilised as a substrate and lowest at rest when glycogen has been resynthesised and is abundant [3–5]. Therefore, a long-standing assumption pervades that AMPK directly interacts with glycogen, presenting a mechanism linking AMPK activation with exercise [2]. This review will present the correlative data that supports the suggestion but, importantly, will highlight the direct evidence that the relationship does not exist in mammalian skeletal muscle *in vivo*.

Mammalian AMPK is an obligate heterotrimeric complex, comprising α, β and γ subunits, for which each has two (α1, α2 and β1, β2) or three (γ1, γ2, γ3) isoforms (for review of AMPK structure, see [6]). Through the possible combinations of these 7 subunits, there are 12 different AMPK heterotrimers that are differentially expressed in various tissues. Of interest in this review, three heterotrimers are found in human skeletal muscle, α1β2γ1, α2β2γ1, and α2β2γ3, with α2β2γ1 being the most abundant and α2β2γ3 the most active form [7]. Once activated, AMPK targets several key downstream targets and processes that synergistically balance energy production and consumption. These processes include, but are not limited to: phosphorylating various metabolic enzymes that inhibit energy utilisation; activating PGC1-α, the transcriptional coactivator that regulates gene transcription to stimulate mitochondrial biogenesis; stimulating protein degradation pathways such as autophagy and the proteosome system; and inhibiting the protein synthesis and cell growth mTOR pathway [1].

Each AMPK subunit has a specific function, which collectively contributes to the cellular interaction activities of the enzyme complex, influencing the enzyme’s sensitivity to changes in AMP and ADP.

### AMPK-α subunit (α-AMPK)

**Figure 1:**
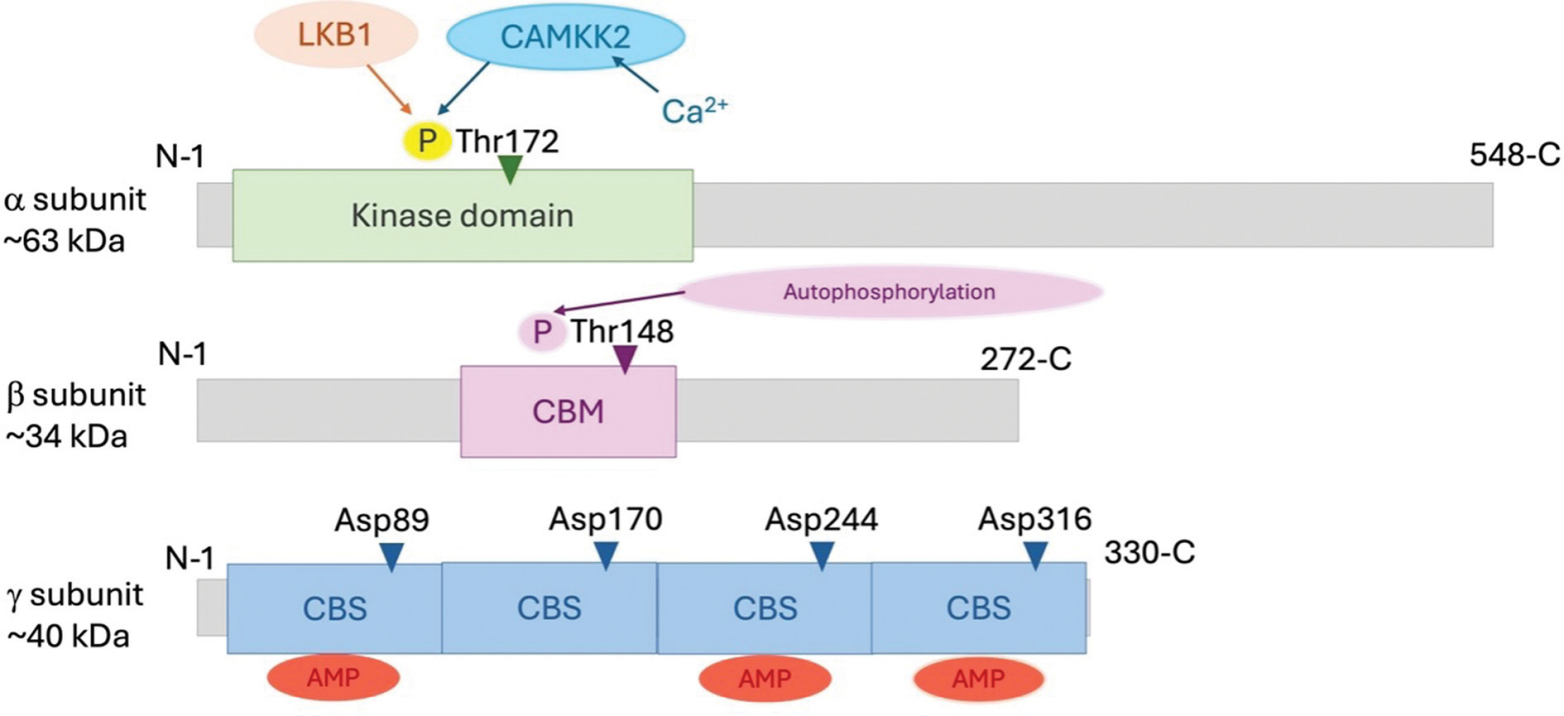
Schematic diagram of heterotrimeric AMPK with pertinent sites discussed in this review indicated The generic subunits are shown, α, β, γ, with acknowledgement that some differences between isoforms exist. The number of amino acids shown from the N-terminus to the C-terminus, and the approximate molecular weights indicated in kDa. The kinase domain of α-AMPK contains the Thr172 site, which can be phosphorylated by upstream kinases, LKB1 or CAMKK2, the latter being regulated by intracellular Ca^2+^ levels. The carbohydrate binding module (CBM) of β-AMPK includes the autophosphorylation site, Thr148. The four cystathionine β-synthase (CBS) motifs are shown on γ-AMPK, with the crucial aspartate (Asp) amino acids that within 3 CBS motifs can bind nucleotides, with the activating AMP shown here. Abbreviations: Asp, aspartate; C, C-terminal; CBM, carbohydrate binding module; CAMKK2, Ca^2+^-calmodulin kinase kinase 2/β; CBS, Cystathionine β-synthase; LKB1, Liver kinase B1; N, N-terminal; Thr, threonine.

The α-AMPK subunit contains the catalytic domain of the AMPK heterotrimer as it contains the kinase domain (Figure 1). AMPK is phosphorylated at Threonine-172 (Thr172) by upstream kinases, predominantly liver kinase B1 (LKB1; [8–10] and calcium/calmodulin-dependent protein kinase kinase 2/β (CaMKK2/β) [11–13] for its activation. There are multiple other α-AMPK phosphorylation sites that have been identified [14], however little is known about whether they are involved in the regulation of AMPK.

### AMPK-β subunit (β-AMPK)

Whilst β1-AMPK is widely expressed in various tissues throughout the body, β2-AMPK is the major isoform responsive *in vivo* in human skeletal muscle [7]. β-AMPK subunits (β1 and β2) play a role in scaffolding the α- and γ- subunits together and each have a carbohydrate-binding module (CBM) (Figure 1), suggesting that AMPK localises with polysaccharides. Indeed, β-AMPK binds carbohydrate and/or glycogen *in vitro* [15] and in cultured cells [16] and AMPK shows µM affinity for glycogen mimetics, such as cyclodextrin [17,18], but these studies do not represent an *in vivo* physiological setting.

The β-AMPK has a phosphorylation site at threonine-148 (Thr148), which is centrally located in the CBM [19] (Figure 1). It was determined that Thr148 of β-AMPK was autophosphorylated and *in vitro* assays revealed that when phosphorylated at Thr148, β-AMPK could not bind to carbohydrate [19]. Importantly, phosphorylated Thr148 (pThr148) β2-AMPK does not associate with glycogen in rat skeletal muscle [20]. Further, the entire measurable pool of β2-AMPK immunoprecipitated with an anti-pThr148 antibody and there was no change in this amount of pThr148 β-AMPK with muscle contraction, which was sufficient to activate AMPK [20].

### AMPK-γ subunit (γ-AMPK)

The γ-AMPK subunits (γ1, γ2 and γ3) contain multiple nucleotide binding sites (CBS domains, Figure 1), responsible for sensing changes in energy status of the cell. γ-AMPK senses the relative levels in AMP and ADP to ATP, which bind competitively to a single site on the γ subunit [21–23]. With AMP or ADP present, γ−AMPK is allosterically activated to induce a conformation change [24], which then confers regulation of AMPK activation through phosphorylation of α-AMPK by its upstream kinases [8,9].

### AMPK-activity

AMPK activity is inhibited by ATP and activated allosterically by AMP, which is required for phosphorylation of Thr-172 of α-AMPK (pThr172) and hence for its activation in the cell [25]. The γ-AMPK subunits detects changes in energy stress, whereby a reduction in ATP coincides with a rise in ADP and AMP, albeit transiently as ATP is rapidly replenished by substrate-level phosphorylation, such as via the action of creatine kinase (ADP + CrP → ATP + Cr), adenylate kinase (2ADP → ATP + AMP) or glycolysis, and then more slowly via oxidative phosphorylation in the mitochondria, and AMP is further degraded to inosine monophosphate. With AMP at the allosteric site, phosphorylation of Thr172 α-AMPK can occur and is commonly used as the indicator of AMPK activation and phosphorylates downstream pathways. One of the first downstream processes is the phosphorylation of acetyl-CoA carboxylase (ACC) at Ser 79, which suppresses malonyl CoA content, which in turn suppresses fatty acid synthesis, removing the inhibition on carnitine palmitoyltransferase (CPT1) which then drives fatty acid oxidation via mitochondrial β-oxidation, and hence increases ATP supply. Phosphorylation of the β-AMPK subunit has been shown to be independent of pThr172 α AMPK [19]. This concurs with the absence of a change in the level of β-AMPK phosphorylation with muscle contraction in rat skeletal muscle [20].

### AMPK localisation in skeletal muscle

**Figure 2:**
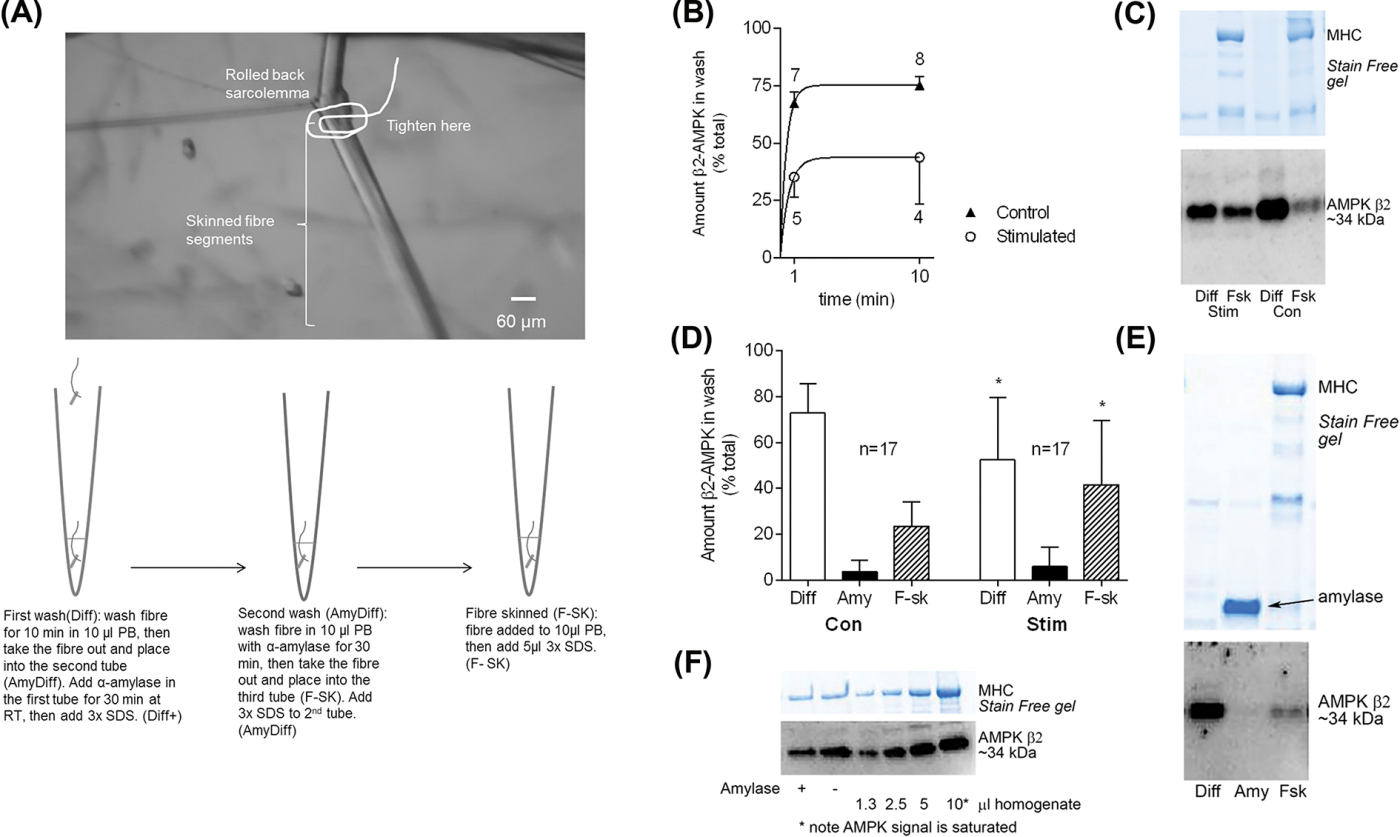
A pool of β2-AMPK becomes bound in rat fast-twitch muscle fibres following fatiguing *in vitro* stimulation, but the bound β2-AMPK is not associated with glycogen (**A**) Shown is a single rat EDL muscle fibre that was mechanically-skinned under paraffin oil to roll back the surface membrane (sarcolemma) and prepared to isolate glycogen pools using amylase treatment. The schematics below detail the various steps involved in collecting the first wash (Diff, Step 1), amylase wash (AmyDiff or Amy, Step 2) and the remaining skinned fibre (F-Sk, Step 3). (**B**) shows pooled data of the diffusibility of β2 AMPK in fast-twitch muscle fibres from control and stimulated EDL muscle with the raw data in (**C**), showing Western blot (bottom) and total protein gel (Stain Free gel, top) indicating the amount of β2-AMPK in the diffusible fraction (Diff) and that remaining in the fibre (F-sk) following a 10 min wash, where there was no amylase step from (A). (**D**) shows pooled data showing β2-AMPK in 1st wash (Diff), glycogen associated (Amy) and remaining in fibres (F-sk) in fast-twitch muscle fibres from control and stimulated EDL muscle after the fatiguing *in vitro* stimulation with the representative western blot and total protein gel (Stain Free gel) shown in (**E**). Panel (**F**) is a Western blot (bottom) and total protein gel (Stain Free gel, top) showing the amount of β2 AMPK in muscle with (+) or without (-) amylase treatment, along with calibration curve of whole muscle homogenate loaded in the amounts indicated. This indicates that amylase treatment, used to fully degrade glycogen, has no effect on the ability to see the total cellular pool of β-AMPK. All data are means (+ SD). The *n* values indicate the number of fibres analysed. *P<0.05 different from Con, one-way ANOVA. Figures reproduced from [26] (A) and [20] (B–F). ***Abbreviations***: Amy, amylase; Diff, diffusible wash; F-sk, skinned fibre

As a master regulator of skeletal muscle function, it is not surprising that AMPK has multiple cellular locations in muscle. Over two-thirds of the total AMPK pool in skeletal muscle has a cytosolic location under resting basal conditions (Figure 2) [20]. Our laboratory isolated fibre segments from glycolytic and oxidative rat muscles and by mechanically skinning removed their sarcolemma to determine the diffusibility (i.e. cytosolic location) of AMPK following exposure to a physiological buffer for 1 and 10 min (i.e., ‘wash’). Regardless of muscle fibre type, the diffusibilities of both β1- and β2-AMPK subunits were similar, with ∼60% of the respective total AMPK protein pools appearing in the wash after 1 min, which increased to ∼75% of the total pools following 10-min washes [20]. Following glycogen utilisation invoked with *in vitro* stimulation, comparisons of fibres from the stimulated and contralateral control muscles found that a pool of β2-AMPK became bound, as the amount of freely diffusible β2-AMPK decreased from ∼75% of the total pool in fibres from control muscles to ∼55% in fibres from stimulated muscles (Figure 2). Most importantly, in various experiments, the AMPK localisation was not influenced by treatment with amylase, which unmasks the entire glycogenin pool [26] and increased appearance of the glycogen debranching enzyme [20], verifying the glycogen granules had been degraded by the amylase. In these experiments, the volume of solution used was ∼1000-fold higher than the fibre volume meaning that the chance of any AMPK released being able to find a new binding partner is very low. Further, using that same methodology, glycogen associated proteins were shown to differentially associate with glycogen following glycogen utilising contractions [27]. Using confocal immunohistochemistry, AMPK has been shown to localise to the nucleus in human [28] and rodent [29] muscle, however, in those studies, it was not possible to determine how much of the total cellular pool is localised in the specific regions due to the lack of calibration and the likelihood of a non-proportional relationship between protein content and output fluorescence.

For more thorough reviews of AMPK in skeletal muscle and exercise, the reader is referred to [30–32].

## Glycogen in skeletal muscle

Glycogen plays a crucial role in skeletal muscle, being the primary source of stored energy available for muscle contraction and other energy utilising processes. Skeletal muscle constitutes ∼40% of total body weight and stores ∼80% of the body’s glycogen. The remainder of the body stores are in the liver, which, relative to its mass, stores a high proportion of glycogen [33,34] with smaller proportions in the brain and heart.

### Glucose uptake into muscle via GLUT4

Homeostatic regulation of blood glucose is imperative for human health. It serves as a major fuel source for energy requirements through the replenishment and maintenance of ATP, as physiological processes demand [35,36]. In skeletal muscle, glucose uptake into the cells (fibres) is via the glucose transporter type 1 (GLUT1) for basal glucose uptake and GLUT4, which mediates the facilitated diffusion of glucose from the bloodstream into muscle, where an increase of insulin (i.e. post-feeding) and/or specific muscle contraction in humans (i.e. exercise) enhance glucose transport into muscle fibres [37–40]. Once inside the muscle, glucose is captured as it is quickly phosphorylated to glucose-6-phosphate via hexokinase. This is the first committed step for using glucose for energy production from where it can be used directly for ATP replenishment via substrate level or oxidative phosphorylation or it can be preserved in its storage form, glycogen.

### Glycogen structure and function

Glycogen is a carbohydrate containing thousands of glucose monomers [41,42]. Via a combination of α1,4-linked bonds and branched by α1,6-linked bonds, the glucose monomers become compact glycogen granules. Glycogen is one of the main fuel sources in skeletal muscle for both short-term and prolonged, repetitive contractions [43] providing an accessible source of ATP during skeletal muscle contraction [44,45]. Three distinct pools of glycogen have been described, intramyofibrillar, intermyofibrillar and sub-sarcolemmal, each likely providing spatially dependent substrate [46]. Glycogen associates dynamically with various proteins [27,47,48] in particular those associated with its synthesis and degradation in skeletal muscle [27]. We have reported that the extent of glycogen association of the glycogen associated proteins differ between resting muscle and following glycogen-utilising contraction of rat skeletal muscle [26,27].

## Exercise

### Contraction types and exercise modalities

Glycogen utilisation during exercise varies based on chronic factors like diet and training status, and acute factors such as the type, duration, and intensity of muscle contractions. Muscle contractions can be simply described as concentric, isometric or eccentric, which involve the sarcomeres inside the muscle fibres either shortening, no change in length, or lengthened, during the contraction, respectively. For example, considering the large *vastus lateralis* muscle (thigh muscle) in humans, bike riding involves predominantly concentric contractions, a wall-sitting exercise involves isometric contractions, and walking or running downhill and resistance exercise involve predominantly eccentric contractions. Importantly, skeletal muscles possess the unique ability to increase energy consumption more than 100- to 300-fold in the immediate shift from rest to exercise (i.e. milliseconds) [49,50], and this ability to adapt to altered energetic demands is essential to skeletal muscle performance.

AMPK activation has typically been associated with endurance exercise; however, it has become increasingly evident that short duration, high intensity exercise is also sufficient to activate AMPK and to see downstream consequences of muscle adaptation to the intervention, such as increases in mitochondria and GLUT4, although when exercise training is addressed, the duration of the training is a variable to consider [5,51–54]. Further, AMPK activation has also been shown to occur following resistance exercise in humans [55] and rats [56].

### AMPK activation with exercise

It is evident that studies have provided a link between exercise, glycogen utilisation and resynthesis, and AMPK activation, and that these factors are likely dependent on both exercise intensity and duration, and muscle fibre type composition. Human skeletal muscle is heterogeneous in nature, being comprised a mixture of slow-twitch, oxidative; fast-twitch oxidative; and fast-twitch glycolytic fibres, typically classified based on the myosin heavy chain (MHC) isoform present. Type I, Type IIA and Type IIX fibres are located side by side in the widely studied *vastus lateralis* muscle and they differ in both metabolic and contractile properties, aptly named as fast or slow according to the speed they contract and relax. Independent studies in humans have measured both whole muscle as well as fibre specific changes following acute bouts of either moderate continuous exercise or high-intensity interval exercise [52,53]. Fibre type specific responses were observed for glycogen utilisation and p172 α-AMPK, which were both greater in type II fibres following an acute bout of high intensity interval exercise [53]. Downstream AMPK activated pSer221 ACC phosphorylation was increased in both Type I and Type II fibres [52,53], with a small but significantly greater increase in Type II compared with Type I fibres following the interval exercise seen in one study [53]. Notably, the fibre type differences in AMPK activation could not be seen using immunofluorescence [57], likely due to the inability to calibrate such systems. Glycogen content, utilisation and resynthesis are both fibre type dependent in human skeletal muscle, albeit dependent on exercise [58,59].

### Glycogen, fasting and exercise

During periods of fasting, liver glycogen is utilised to maintain blood glucose homeostasis. Muscle glycogen cannot contribute to this homeostatic regulation as muscle does not contain glucose-6-phosphatase (G6P), which is necessary to convert G6P back to glucose. During exercise of a duration and intensity where the requirement for energy cannot be met by circulating glucose, muscle glycogen is utilised [59,60]. One of the seminal studies in exercise physiology showed that with strenuous exercise, where glycogen utilisation neared depletion, consumption of high carbohydrate foods resulted in higher intramuscular glycogen than was previously present [61]. This is known as glycogen supercompensation and is routinely used by athletes to ensure high glycogen stores prior to strenuous exercise events [62].

## Glycogen and AMPK in skeletal muscle

### Glycogen and AMPK association

**Figure 3:**
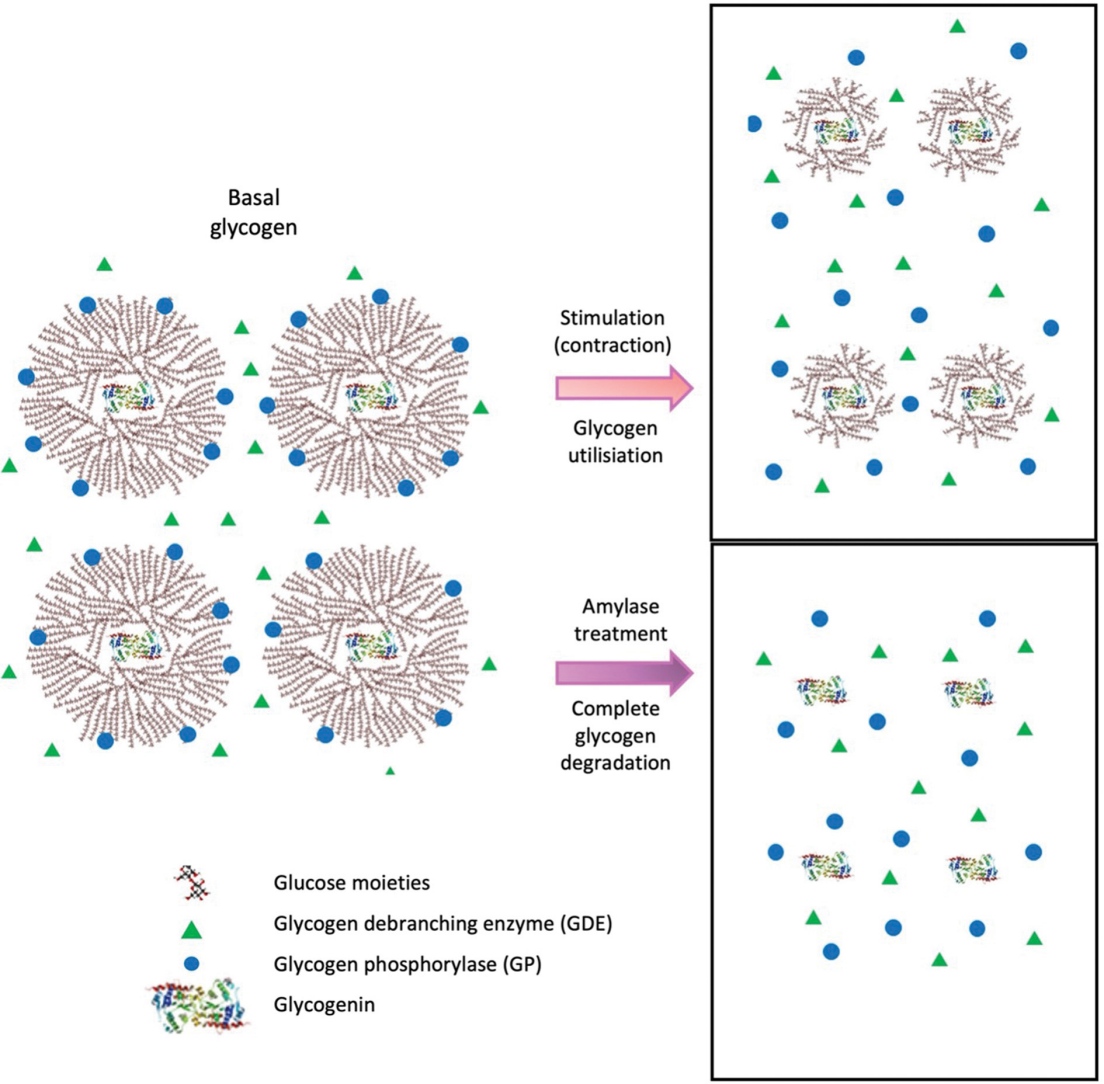
Glycogen granules in skeletal muscle Schematic of glycogen granules in resting skeletal muscle (basal). Following degradation via glycogen utilising muscle stimulation (contraction), the outer glucose moieties are removed being released as the α-1,6 linked bonds are cleaved via the action of glycogen debranching enzyme (GDE) and the α-1,4-linked bonds via the action of glycogen phosphorylase (GP). Glucose is used and GDE is no longer attached to the remaining smaller glycogen granule. Following complete glycogen degradation via amylase treatment, all glucose moieties are released along with glycogen associated proteins as shown by the release of the core glycogen protein, glycogenin. Adapted from [26]. Abbreviations: GDE, glycogen debranching enzyme; GP, glycogen phosphorylase

There is solid biochemical evidence that purified AMPK can bind to carbohydrate moieties *in vitro* [15,17]. AMPK was described as associating with hepatic glycogen particles in liver as assessed using electron microscopy [63], however using alternate biochemical techniques, including assessing the glycogen proteome, western blotting and measuring β1-AMPK CBM affinity for sucrose (i.e. a carbohydrate disaccharide) using fluorescence in liver and muscle tissue, no association of AMPK with glycogen could be ascertained [48]. Using a mechanically-skinned muscle fibre preparation, neither α-AMPK nor β-AMPK were found to appreciably associate with the amylase digestible components of the muscle, that is, the compartment containing glycogen particles following degradation of the glycogen phosphorylase α-1,4 linkages [27]. This was also the case in muscle stimulated *in vitro* under differing states of glycogen utilisation and using amylase to degrade the glycogen granules and release glycogen bound proteins, such as glycogenin and glycogen debranching enzyme (Figure 3) [26].

Whether an *in vivo* association exists was further examined by considering the CBM domain of β-AMPK biochemically in an *in vitro* system, which demonstrated that auto phosphorylation of Thr148 β-AMPK mediates a loss in the ability for AMPK to bind carbohydrate moieties [19]. When investigated in a more physiological setting of rat skeletal muscle, it was shown that β2-AMPK is intrinsically phosphorylated at Thr148 [20]. These data strongly suggest that AMPK does not associate with glycogen in skeletal muscle and adopting a mechanically-skinned muscle fibre technique, little AMPK was found to be associated with the glycogen rich pool [20]. Using the same mechanically skinned muscle fibre technique, glycogen associated proteins could variably be seen in the skeletal muscle glycogen pools demonstrating the robustness of this method [27].

Despite the lack of evidence for a direct AMPK-glycogen association, others have performed studies in a mutant mouse model claiming to have a ‘glycogen binding’ mutation that disrupts the glycogen binding capacity of AMPK [64]. Critically, however, these authors provided no evidence to demonstrate that endogenous AMPK was indeed bound to glycogen in WT animals, which would counter the finding by our laboratory [20], and this is imperative to the correct interpretation of the data.

### Is AMPK a direct glycogen-sensor in skeletal muscle?

Given the correlation between exercise, glycogen utilisation and AMPK activation [3,53,65] and the need to increase glucose uptake into the muscle fibres both during and post exercise, it is tempting to suggest that glucose uptake is triggered as a consequence of glycogen utilisation and that AMPK directly senses this via an association with glycogen. As acknowledged many years ago [65], such data are correlative and does not support causation of glycogen regulating AMPK activity. Over years of research, evidence has been documented to suggest AMPK does bind to glycogen, however, as outlined here, they are typically within an *in vitro* setting. With methodological advances and revisiting of this question, the relevance of those findings to mammalian skeletal muscle can now be considered.

For instance, it was shown many years ago that AMPK coimmunoprecipitated with glycogen synthase (GS) in liver [66] and muscle [67] preparations. Whilst some of the total pool of GS resides with glycogen granules, this amounts to only ∼50% of the total GS pool in rat skeletal muscle [27]. As such, the presence of GS and AMPK in the same biochemical pool is not evidence that AMPK is bound to glycogen. Suggestions that AMPK is acting as a type of ‘glycogen sensor’, with AMPK being released from the glycogen-bound pool as glycogen is degraded is not supported because AMPK becomes further bound in skeletal muscle with muscle contraction and does not become more freely diffusible, which must be the consequence if glycogen degradation resulted in ‘freeing bound’ AMPK [20].

Importantly, the Thr148 β-AMPK phosphorylation site is within the CBM of β-AMPK and when phosphorylated is prevented from binding to carbohydrate *in vitro* [19]. Using rat skeletal muscle, pThr148 β-AMPK is intrinsically phosphorylated as coimmunoprecipitation experiments with an anti- pThr148 β-AMPK antibody resulted in the entire pool of AMPK being isolated with the coimmunoprecipitate and none in the remaining supernatant, and this did not change with glycogen utilising muscle contractions [20]. This means that β-AMPK cannot bind to glycogen in rodent skeletal muscle.

AMPK is a master regulator of metabolism, responding to periods of low energy or ATP availability, which is sensed as increases in downstream nucleotides, in particular AMP. The fact that skeletal muscle AMPK is activated and inhibited during periods of low and high glycogen, which occur in states of muscle use and rest, respectively, has no empirical evidence demonstrating causation. Indeed, in real world scenarios like McArdle’s Disease, where patients lack the enzyme glycogen phosphorylase required to degrade a1,4-linked bonds, glycogen levels remain high and AMPK is activated during exercise, despite the absence of glycogen breakdown [68]. First most, any such studies must prove that there is association of AMPK with glycogen in skeletal muscle which is then reduced, which to date have not been forthcoming.

The CBM on β-AMPK is widely conserved throughout eukaryotes and AMPK can bind carbohydrates in cell-free systems yet is not able to bind to glycogen in skeletal muscle. Cell-free carbohydrate binding assays revealed that phosphorylation at Thr148 β-AMPK abolished the capacity to bind carbohydrate and subsequently it has been shown that the entire pool of β-AMPK in rodent skeletal muscle is phosphorylated at Thr148. Thus, the scientific evidence to date shows the importance of using physiological settings to determine the relevance of post-translational modifications, which affect function, and in the case of β-AMPK, its capacity to bind endogenous carbohydrate, that is glycogen, despite it containing a CBM. Given the lack of association evidence to date, the changes in skeletal muscle AMPK and glycogen are correlative and not causative for downstream activity.

## Summary

- AMPK is considered a master regulator of skeletal muscle metabolism.
- Functional and metabolic demands of skeletal muscle result in glycogen utilisation and AMPK activation.
- Current thinking is that AMPK binds to glycogen and that glycogen utilisation directly results in AMPK activation.
- AMPK, however, does not bind to glycogen in skeletal muscle *in vivo.*
- Hence, glycogen does not directly regulate AMPK activity in skeletal muscle.

## Open Access

Open access for this article was enabled by the participation of La Trobe University in an all-inclusive *Read & Publish* agreement with Portland Press and the Biochemical Society under a transformative agreement with CAUL.

## Abbreviations

ACC: acetyl-CoA carboxylase
ADP: adenosine diphosphate
AMP: adenosine monophosphate
AMPK: 5′-AMP-activated protein kinase
ATP: adenosine triphosphate
CaMKK2/β: calcium/calmodulin-dependent protein kinase kinase 2/β
CBM: carbohydrate-binding module
CBS: cystathionine β-synthase motif
CPT1: carnitine palmitoyltransferase
Cr: creatine
CrP: creatine phosphate
F-sk: skinned fibre
G6P: glucose-6-phosphate
GLUT1: glucose transporter type 1
GLUT4: glucose transporter type 4
GS: glycogen synthase
LKB1: liver kinase B1
MHC: myosin heavy chain
mTOR: mammalian target of rapamycin
PGC1-α: peroxisome proliferator-activated receptor gamma coactivator 1-α
